# Clinical outcomes of chlormethine gel in mycosis fungoides affecting “sensitive” areas: A retrospective case series

**DOI:** 10.1111/ddg.15797

**Published:** 2025-06-29

**Authors:** Gianluca Tavoletti, Gianluca Avallone, Pamela Vezzoli, Paolo Sena, Angelo V. Marzano, Emilio Berti, Silvia Alberti‐Violetti

**Affiliations:** ^1^ Dermatology Unit Fondazione IRCCS Ca' Granda Ospedale Maggiore Policlinico Milan Italy; ^2^ Department of Pathophysiology and Transplantation Università degli Studi di Milano Milan Italy; ^3^ Dermatology Unit ASST Papa Giovanni XXIII Bergamo Italy; ^4^ Inter‐Hospital Pathology Division IRCCS MultiMedica Milan Italy

**Keywords:** chlormethine, cutaneous lymphomas, Mycosis fungoides

Dear Editors,

Chlormethine (CL) gel is the first skin‐directed therapy developed for the treatment of mycosis fungoides (MF).^1^ It is recommended as a first‐line treatment for adult patients with early‐stage MF (IA–IIA) and, in combination with systemic therapies, for those in advanced stages, according to international guidelines.^2^, ^3^, ^4^ Although clinical trials and real‐world evidence support its efficacy,^5^, ^6^ application of CL gel to anatomically “sensitive” areas (e.g., face, anogenital, and intertriginous regions) remains challenging due to the risk of local adverse events (AEs), particularly irritant contact dermatitis (ICD).^7^ While some anecdotal evidence suggests that CL gel may be used in such sites,^8^, ^9^ there is a need for real‐world data to elucidate its effectiveness and to detail management strategies for associated AEs.

We conducted a retrospective case series at two Italian tertiary referral centers from January 2020 to April 2024. The study was approved by the Ethics Committee Milano Area 2 (Protocol No: 0007202). Eligible patients had a histologically confirmed diagnosis of MF with at least one “sensitive” area involved and were treated with CL gel. Assessments were performed at baseline and every 3 months, in accordance with standard clinical care and institutional protocols. The response was evaluated using a *modified Composite Assessment of Index Lesion Severity* (mCAILS), which excluded hyperpigmentation.^7^, ^8^ The objective response rate (ORR), defined as the proportion of patients who achieved either a complete (mCAILS = 0) or partial response (≥ 50% reduction in mCAILS) at the three‐month evaluation,^6^ was calculated. AEs were graded according to the *National Cancer Institute Common Terminology Criteria for Adverse Events, version 5.0*.^10^

We included eight patients (5 males and 3 females) at different stages of MF (75% in early stage) (Table 1). The median age of patients was 56.5 years (Q1 = 50; Q3 = 58.5), and the median age at diagnosis was 43.5 years (Q1 = 40; Q3 = 49). Clinical variants were classical MF (n = 5), folliculotropic MF (n = 2), and poikilodermatous MF (n = 1). “Sensitive” areas involved the eyelid (n = 3), groin, cheek, perineum, pubic region, penis, and scrotum. Four patients (50%) used concomitant topical corticosteroids (TCS) to reduce local irritation, and three (38%) were receiving systemic therapy. Two patients (25%) applied CL gel daily, and six patients (75%) applied it on alternate days. The median treatment duration was 3 months (Q1 = 2; Q3 = 3.5). At three months, six patients (75%) achieved the ORR, while two had persistent disease, yet decreased in severity. Among patients undergoing systemic therapy, CL gel was introduced on targeted recalcitrant or new lesions, further supporting overall disease control. At a median follow‐up of 22 months (Q1 = 14; Q3 = 42), six patients achieved and maintained a complete response (mCAILS = 0), while two (25%) displayed minimal residual disease (Figure 1). Adverse events occurred in six patients (75%) and included mild ICD in three patients (38%), moderate ICD in two (25%), and severe ICD in one (13%). All but one patient continued CL gel after a brief interruption or a reduced application frequency. Adjunctive TCS was used to manage inflammation, with clinicians remaining cautious due to the heightened risk of steroid‐induced atrophy in sensitive areas. Transient hyperpigmentation was observed in the treated areas of six patients (75%).

**TABLE 1:** Summary of patient characteristics.

Patient No.	1	2	3	4	5	6	7	8
Age/sex	46/M	57/M	42/M	81/M	59/F	54/F	56/F	58/M
Age at MF diagnosis (years)	38	42	19	68	46	48	50	43
Fitzpatrick skin type	II	III	III	III	III	III	III	II
Clinical variants	Classical MF	Poikilodermatous MF	Classical MF	Classical MF	Folliculotropic MF	Classical MF	Folliculotropic MF	Classical MF
Prior MF therapy	TCS, nb‐UVB, aci	TCS, PUVA, nb‐UVB,	TCS, aci,	TCS, nb‐UVB, RT	TCS, PUVA, aci, bexa, ifn, RT	TCS, PUVA, aci, RT	TCS, nb‐UVB, aci, PUVA	TCS, nb‐UVB, aci
Age at CL introduction	44	40	41	80	55	50	54	57
Stage at CL introduction	Ia	Ia	Ib	Ib	IIb	Ib	IIb	IIa
Affected sensitive area	Groin	Perineum	Eyelid	Pubis	Eyelid	Eyelid	Cheek	Penis and scrotum
Type of lesion treated	Patch	Plaque	Patch	Plaque	Plaque	Plaque	Plaque	Plaque
Frequency of CL application	Every other day	Every other day	Every day	Every other day	Every other day	Every day	Every other day	Every other day
Duration of CL treatment	3 months	4 months	5 days	2 months	3 months	9 months	3 months	2 months
Use of combination topical therapies	TCS	TCS	None	TCS	None	None	None	TCS
Use of combination systemic therapies	None	None	None	None	Bexa	none	PUVA	Aci
mCAILS at CL introduction (T0)	16	20	11	22	12	13	15	11
mCAILS at T3	7	9	0	0	0	8	2	6
mCAILS at T6	0	0	0	0	0	5	0	3
mCAILS at T12	0	0	0	0	0	3	0	3
Months to last follow‐up	36	14	14	24	48	48	24	12
mCAILS at the last follow‐up	0	0	0	0	0	3	0	3
Hyperpigmentation	Yes	Yes	Yes	Yes	Yes	No	Yes	No
Adverse events	ICD (mild)	ICD (moderate)	ICD (severe)	ICD (moderate)	ICD (mild)	None	None	ICD (mild)
Management of adverse events	Use of TCS	Temporary stop	Stop, use of TCS	Temporary stop, reduced dosing frequency, use of TCS	Temporary stop	None	None	Reduced dosing frequency

*Abbr*.: TCS, topical corticosteroids; nb‐UVB, narrow‐band ultraviolet B; PUVA, psoralen and ultraviolet A; RT, radiotherapy; aci, acitretin; bexa, bexarotene; ifn, interferon; mCAILS, modified Composite Assessment of Index Lesion Severity; ICD, irritant contact dermatitis; MF, mycosis fungoides; CL, chlormethine

**FIGURE 1:**
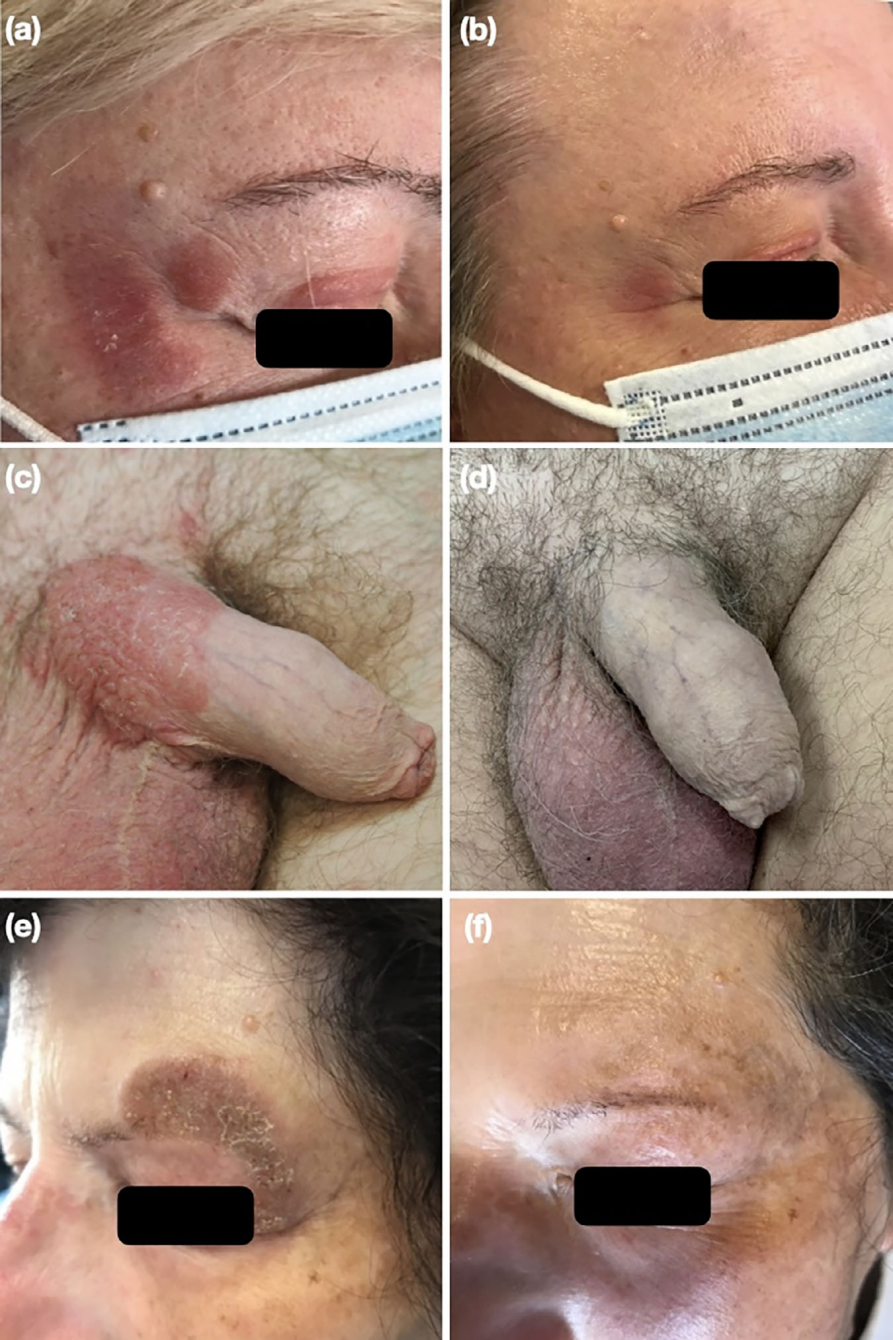
Patients with mycosis fungoides in sensitive areas treated with chlormethine gel: (a, c, e) baseline appearance and (b, d, f) appearance at the last follow‐up.

Although clinical trials suggest that sensitive areas are at increased risk of AEs related to CL gel use,^7^, ^8^ this retrospective case series demonstrates that CL gel can be both safe and effective for MF lesions in sensitive skin regions. The concomitant use of TCS appears to enhance tolerability, aligning with prior observational evidence.^6^

Study limitations include the retrospective design, the relatively small sample size, and the exclusive inclusion of Caucasian patients. Future prospective studies should investigate optimal application schedules, evaluate supportive measures to prevent or manage ICD, and further clarify the role of CL gel in combination with systemic agents.

## CONFLICT OF INTEREST STATEMENT

None.
